# FHIR Data Mapping Quality Assessment: A Scoping Review and Expert-Informed Requirements for Future Pre-Mapping Frameworks

**DOI:** 10.34133/csbj.0231

**Published:** 2026-10-22

**Authors:** Mattias Seth, Fanny Apelgren, Anna Bakidou, Peter Lundgren, Hans Blomberg, Bengt Arne Sjöqvist, Stefan Candefjord

**Affiliations:** ^1^ Department of Electrical Engineering, Chalmers University of Technology, SE-412 96 Gothenburg, Sweden.; ^2^ PreHospen—Centre for Prehospital Research, Faculty of Caring Science, Work Life and Social Welfare, University of Borås, Borås, Sweden.; ^3^ Department of Cardiology, Sahlgrenska University Hospital, Gothenburg, Sweden.; ^4^ Center for Digital Health, Sahlgrenska University Hospital, Gothenburg,Region Västra Götaland, Sweden.; ^5^ Department of Thoracic Surgery and Cardiology, Sahlgrenska University Hospital, Gothenburg,Region Västra Götaland, Sweden.; ^6^ Institute of Medicine, Sahlgrenska Academy, University of Gothenburg, Gothenburg, Sweden.; ^7^ Department of Surgical Sciences, Uppsala University, Uppsala, Sweden.

## Abstract

**Background:** Healthcare systems increasingly rely on effective data sharing to support safe and efficient care. The fast healthcare interoperability resources (FHIR) standard provides a widely adopted framework for interoperability. However, many existing healthcare information systems predate FHIR and require translation or mapping of their data models and data elements to the FHIR standard to enable interoperability. Despite growing FHIR adoption, robust approaches for assessing mapping quality remain limited. **Objective:** This scoping review aimed to identify and synthesize existing tools and approaches for evaluating FHIR data mapping quality, examine methodological challenges and gaps, and formulate and refine requirements for more consistent and transparent assessment. **Methods:** A scoping review was conducted across IEEE Xplore, ACM Digital Library, PubMed, and the JMIR journal family. Studies were screened using predefined eligibility criteria and analyzed through thematic synthesis focusing on mapping tools, validation approaches, challenges, and quality assessment dimensions. FHIR mapping quality was conceptualized using 7 quality dimensions adapted from data quality literature. Candidate framework requirements were formulated and refined through expert consultation (*n* = 4). **Results:** A total of 742 studies were screened and 39 included. The literature revealed fragmented practices with limited consensus on FHIR mapping quality assessment. Most studies relied on manual evaluation of the transformed data, while few considered source-to-target relationships before transformation. Six requirements for future pre-mapping quality assessment frameworks were formulated and refined. **Conclusions:** Current FHIR mapping quality assessment approaches remain fragmented and largely rely on manual evaluation. The findings highlight the need for more transparent and formalized pre-mapping assessment approaches to support future FHIR implementations.

## Introduction

At a time when global healthcare systems are constrained by economic pressures, rising multimorbidity, and an aging population [1,2], effective data sharing and use [3,4] are more critical than ever to support clinical decision-making, remote patient monitoring [5], and precision medicine [6] and improve patient safety [7]. However, due to regulatory requirements related to data protection, scalability limitations, established clinical workflows, and legacy technical infrastructures (often relying on proprietary data models), health data are often fragmented and stored in nonstandardized formats across weakly interconnected data silos [8]. As a result, healthcare organizations often face difficulties in accessing and effectively using data [3]. This challenge is illustrated in Fig. 1.

**Fig. 1.**
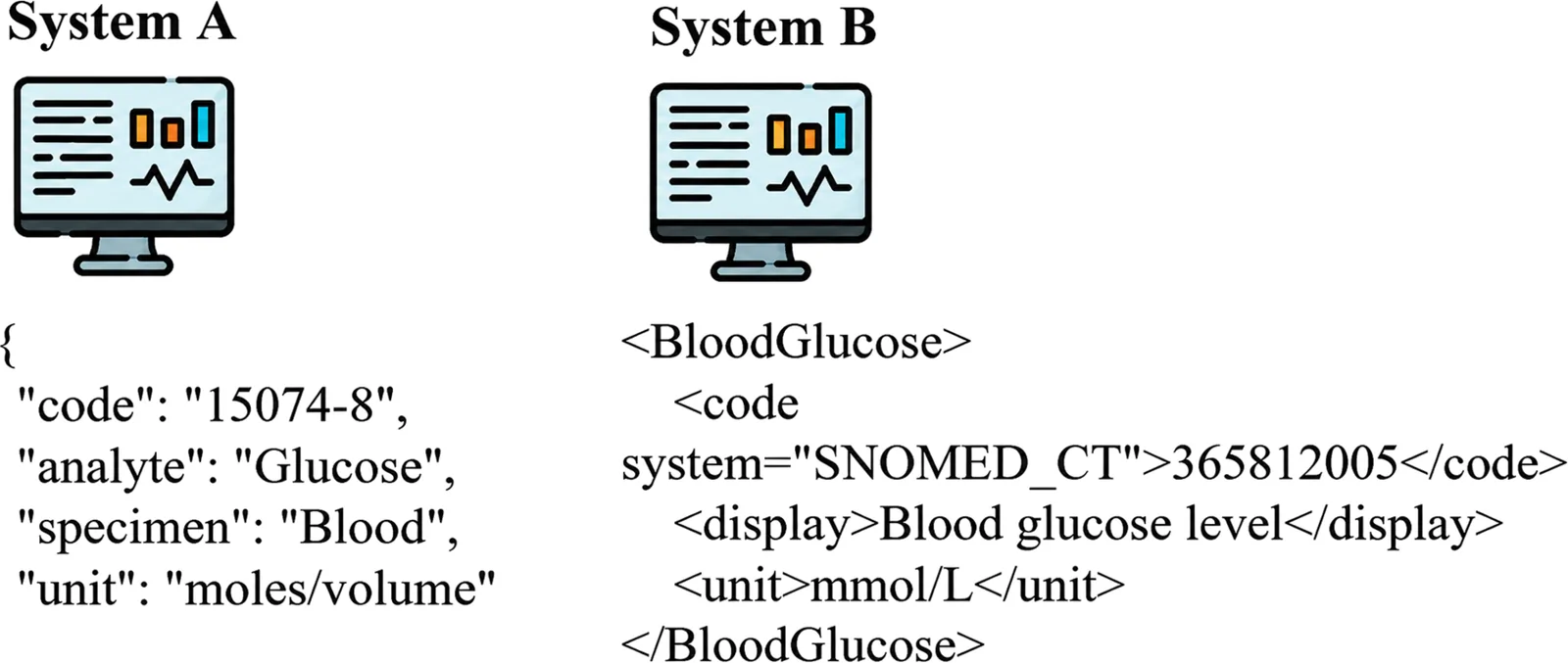
Example of 2 separate systems storing the same type of information using different formats and semantics. System A uses JavaScript object notation (JSON) and logical observation identifiers names and codes (LOINC), while system B uses extensible markup language (XML) and the Systematized Nomenclature of Medicine—Clinical Terms (SNOMED CT). Connecting these systems requires data-mapping strategies.

Although the example in Fig. 1 appears simple, real-world electronic health record (EHR) systems contain thousands of heterogeneous data elements and rely on extensive clinical terminologies such as International Classification of Diseases (ICD) and Systematized Nomenclature of Medicine—Clinical Terms (SNOMED CT) [9]. Consequently, interoperability requires more than semantic translation between terminology systems; it also involves structural mapping between different data models, hierarchies, and message formats [10]. As a result, connecting multiple systems without shared formats and semantics becomes highly complex [11].

To establish the context for this review, the following sections first describe the role of FHIR in enabling healthcare interoperability and the need for data mapping when integrating legacy and heterogeneous systems. We then examine current approaches to FHIR mapping and validation, before outlining the remaining gaps that motivate this review.

### The role of FHIR to achieve interoperability

One approach to address interoperability challenges is the use of standardized data models and exchange mechanisms [11,12]. A leading example is the fast healthcare interoperability resources (FHIR) standard, developed by Health Level Seven International (HL7) since 2011 [13]. FHIR defines modular “Resources” represented in JavaScript object notation (JSON) or extensible markup language (XML) to structure and exchange clinical information consistently across systems [3]. Due to its modern web-based design and strong community support, FHIR has become one of the most widely adopted standards for health data exchange [13], including within major EHR vendors, technology companies [13], and international initiatives such as the European Health Data Space (EHDS) [8,14].

Despite the increasing adoption of FHIR, many healthcare information technology systems were developed long before its introduction and continue to rely on proprietary or non-FHIR-compatible technologies [10]. Achieving interoperability with FHIR-compliant systems therefore often requires data translation, commonly referred to as data mapping. For the systems illustrated in Fig. 1, this includes translation between JSON and XML formats as well as between LOINC (logical observation identifiers names and codes) and SNOMED CT terminologies. The data mapping process is illustrated in Fig. 2 and typically involves 3 steps: (a) identification of source and target datasets (i.e., the original data to be mapped and its intended FHIR representation), (b) specification of mapping rules, and (c) transformation of the data into the target representation.

**Fig. 2.**
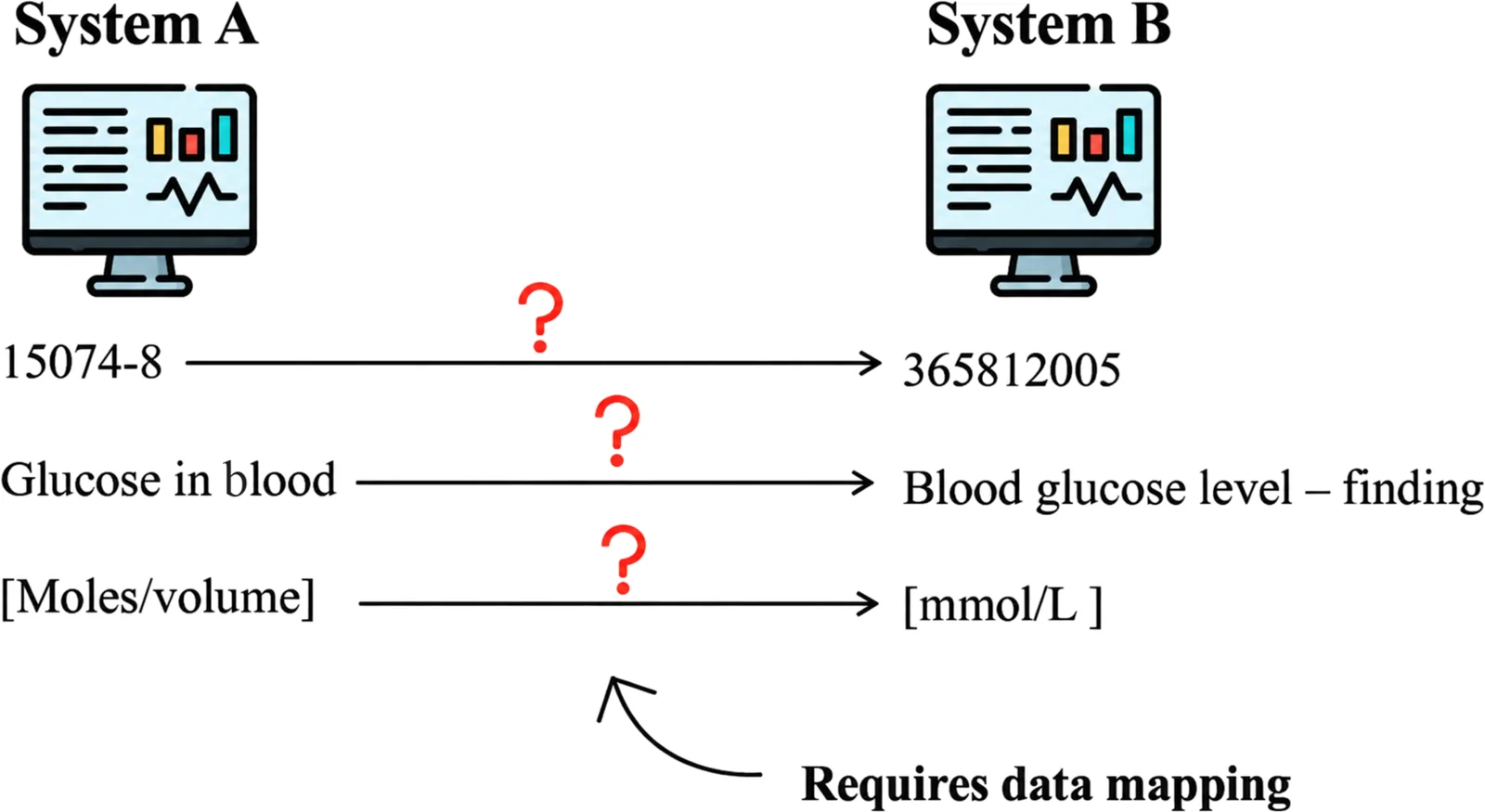
For system B to interpret information from system A, data needs to be mapped. This process typically comprises identification of source and target datasets (systems A and B), specification of mapping rules, and transformation of the data into the target representation.

Data mapping is a prerequisite for interoperability because it enables systems using different syntactic and semantic representations to exchange and interpret information consistently [10,11]. However, mapping processes are often time-consuming, ambiguous, and prone to errors due to the need for substantial manual effort and interpretation by implementers [10,15]. These challenges were recognized by the Care@Distance research team at Chalmers University of Technology during FHIR data mapping for the on-scene injury severity prediction (OSISP) trauma triage model [16]. The mapping process highlighted practical difficulties in aligning trauma variables and artificial intelligence (AI)-related model information with standardized FHIR semantics without introducing interpretation bias. Several variables could reasonably be mapped to multiple FHIR resources, some required data-type transformations, and others required custom extensions. These ambiguities raised practical questions regarding mapping appropriateness, consistency, and validation. Similar challenges have been reported in other studies [14,17,18].

### Current FHIR mapping and validation approaches

To support and simplify the FHIR mapping process, various tools have been introduced by the HL7 community and research groups. One example is the FHIR mapping language (FML), a declarative language used to define transformation rules between source datasets and target FHIR structures through the StructureMap FHIR resource (Fig. 3) [14,19].

**Fig. 3.**
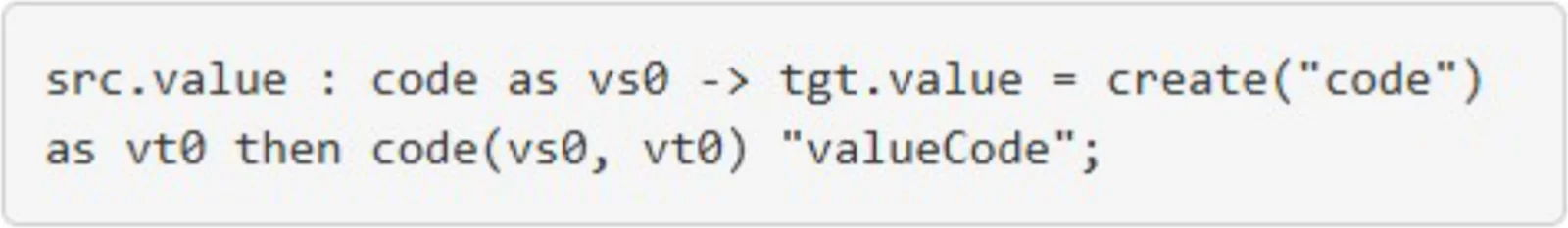
Mapping rule using FML. Each rule has 3 parts: the source content (before “->”), which defines the input elements, and the target transform (after “->”), which defines how the source is used to create the target using FHIRPath expressions. An optional “then” section lists dependent rule groups.

Other approaches use AI, machine learning (ML), and natural language processing (NLP) to automate or semi-automate mapping processes [10]. For example, NLP2FHIR, introduced by researchers at Mayo Clinic, applies NLP techniques to extract structured FHIR resources from unstructured clinical narratives (Fig. 4) [20–22]. Ontology-based approaches have also been proposed to support more systematic and reusable mappings [23].

**Fig. 4.**
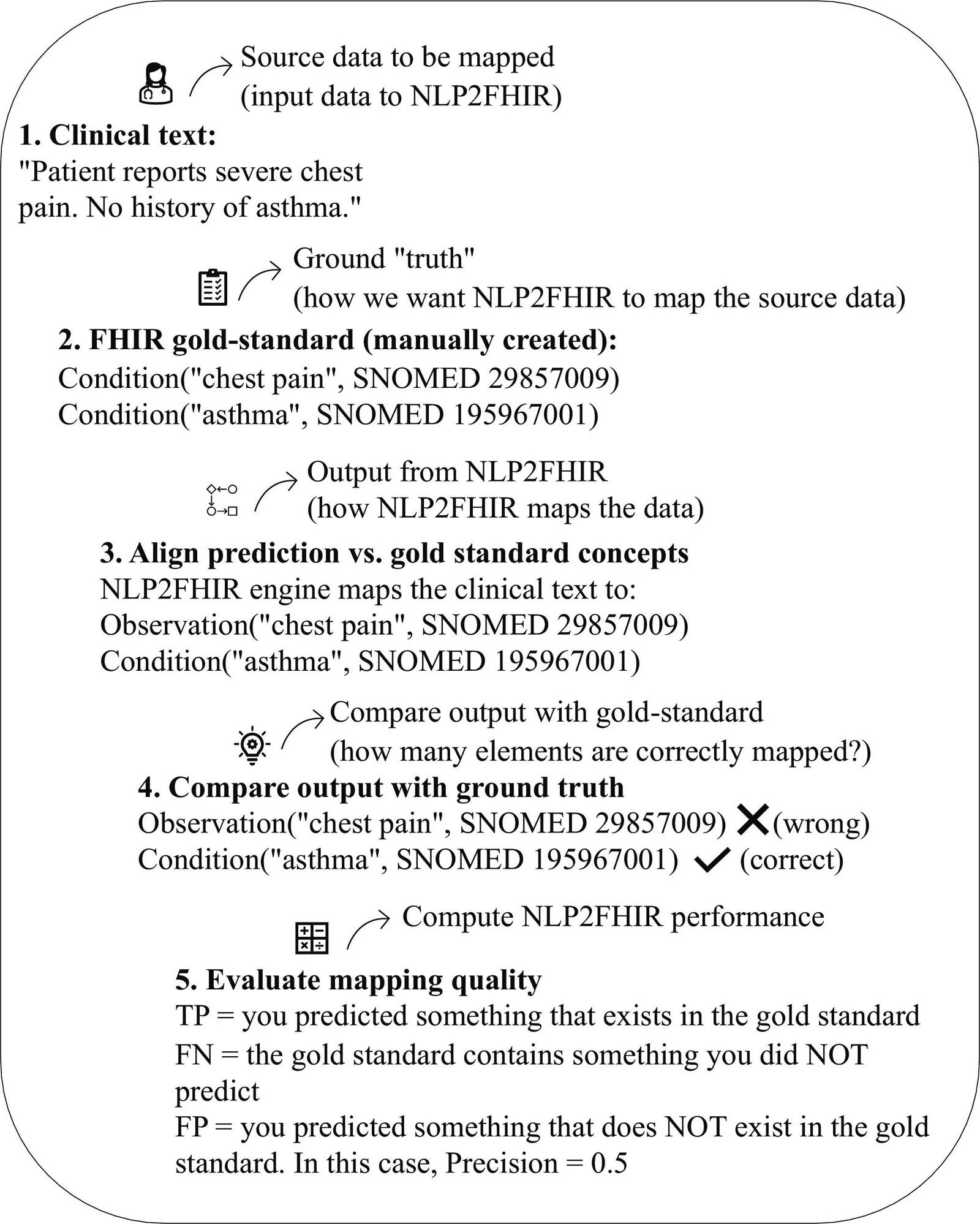
Example of post-mapping quality assessment in NLP2FHIR. Evaluation considers FHIR resource type and concept alignment with the gold standard. The gold-standard dataset (step 2) is created from manually annotated clinical narratives, and evaluation metrics such as true positives (TP), false negatives (FN), and false positives (FP) (step 5) may vary depending on project goals.

Despite these developments, mapping efforts often remain highly dependent on project-specific tools, local implementations, and individual interpretation [15]. Consequently, ambiguous mapping rules may lead to inconsistent and nonreproducible results, undermining interoperability and the intent of standardization. Validation of FHIR mapping quality therefore remains fragmented and inconsistent, frequently relying on manual inspection or post-mapping validation approaches. Such approaches commonly use tools including the HL7 FHIR Validator, Inferno Validator, or Extract, Load, and Transform (ETL) tools to assess whether generated FHIR resources conform syntactically to schemas or FHIR profiles. While these methods are important for verifying structural correctness and profile conformance, they do not necessarily assess whether the target representation accurately reflects the source data. Pre-mapping validation approaches, where datasets and mapping assumptions are evaluated before transformation begins, may help reduce ambiguities and information loss, yet such approaches remain uncommon in practice [24].

#### Identified gaps

Taken together, these limitations highlight a broader gap in how FHIR mapping quality is defined and evaluated, particularly regarding whether assessment should depend primarily on individual expertise or be guided by standardized evaluation criteria. Without clearer approaches for assessing mapping quality, ambiguous and inconsistent mappings risk becoming accepted practice, thereby undermining interoperability and reproducibility.

Therefore, this scoping review aims to identify, categorize, and synthesize approaches used to assess the quality of FHIR data mapping, including both pre-mapping and post-mapping validation strategies. This review not only synthesizes existing approaches but also formulates and refines conceptual requirements for future pre-mapping assessment frameworks.

### Objective

Based on our own experience, and to complement previous research on FHIR data mapping tools, the aim of this scoping review is to synthesize how the quality of FHIR data mappings is currently evaluated and to identify gaps that hinder reliable data use and interoperability. Based on the findings, the ambition is to establish a set of requirements that can inform and guide future initiatives focused on developing a framework for FHIR mapping quality assessment.

To achieve this objective, the following research questions were defined:

1. How is the quality of FHIR data mappings currently evaluated, and what gaps hinder their reliable use and impede interoperability in healthcare systems?
2. Are there any standardized tools or approaches to assess FHIR data mapping quality (both post- and pre-mapping approaches). If not, what key characteristics should an FHIR mapping quality assessment framework possess?

## Methods

This scoping review followed PRISMA-ScR guidelines. A structured literature search identified studies on FHIR data mapping tools, validation approaches, and associated challenges, using predefined search strategies, eligibility criteria, study selection, data extraction, and thematic synthesis. Data were defined as collected facts in any format with potential informational value [25]. The study selection process is illustrated in Fig. 6. No formal risk-of-bias assessment was performed, consistent with PRISMA-ScR guidance.

Rather than treating FHIR mapping quality as a single construct, it was operationalized as 7 dimensions (Table 1) to support the synthesis. These dimensions were not identified as a standardized framework in the literature but were derived and adapted from established data quality literature [25,26] and established data quality frameworks (e.g., ISO 25012 [27]). These dimensions— (a) coverage, (b) completeness, (c) semantic correctness, (d) terminology accuracy, (e) structural conformance, (f) inter-rater reliability, and (g) retrievability (Table 1)— were defined prior to data extraction and used as the initial analytical framework during data extraction and thematic synthesis. Their definitions were subsequently refined iteratively as ambiguities became apparent during comparison across the included studies. Whenever a definition was refined, all included studies were revisited to ensure that the final definitions were applied consistently across the complete dataset. The terms assessment and validation were not consistently distinguished in the included literature. In this review, assessment is used as the broader term for evaluating mapping-related quality, whereas validation refers to specific activities used to verify properties of the mapping or resulting target representation.

**Table 1.** Operational definitions of 7 mapping quality assessment dimensions used in the thematic synthesis. Each quality assessment dimension is associated with potential errors if not evaluated/used correctly.

Quality assessment dimension	Validation of:	Potential errors if not validated:
Coverage	% of source elements with corresponding FHIR elements	Overuse of extensions; missing concepts; unmapped source elements
Completeness	Null values, duplicates, missing required fields	Missing or incomplete required fields; duplicate entries
Semantic correctness	Chosen FHIR resource, placements of attributes and relationships	Wrong resource used; incorrect attribute mapping, i.e., “Unknown” becomes “False”
Terminology accuracy	Value sets, binding strength (required, preferred, extensible), coding pattern (code + system + display)	Incorrect terminology system (e.g., LOINC instead of SNOMED)
Structural conformance	Resource structure, including datatypes, cardinality requirements and slicing	Invalid datatypes (requires conversions); cardinality violations (multiple values where only one is allowed)
Inter-rater reliability	Agreement between multiple mappers or reviewers assigning FHIR resources, attributes, or codes	Inconsistent mappings; subjective variation; reduced reproducibility; biased ground-truth/gold-standard datasets
Retrievability	Ability to search, filter, and retrieve mapped data using standard FHIR search parameters; correctness of query results	Data cannot be retrieved as before; empty search results; incorrect or incomplete query outputs

Each dimension captures a specific aspect of how source data are represented, validated, and reused within the FHIR ecosystem, regardless of data format. Together, they form the analytical framework used to classify validation tools, quality assessment dimensions, challenges, and gaps identified in the included studies. Importantly, the 7 dimensions in Table 1 should not be interpreted as equivalent properties of source-to-target mapping quality. Rather, depending on how they are assessed, individual dimensions may primarily concern the source-to-target transformation, properties of the resulting FHIR representation (target only), reproducibility of the mapping process, or downstream usability of the mapped data. For example, while assessment of coverage, semantic correctness, and terminology accuracy generally requires consideration of the source data and its intended FHIR representation, structural conformance can be assessed on the target data (resulting FHIR representation) independently of the source data.

The dimensions in Table 1 were used to classify quality-related assessment activities reported in the context of FHIR mapping. As described above, these activities may concern either the source-to-target transformation or properties of the resulting FHIR representation. To distinguish these perspectives, a decision model was developed to differentiate source-to-target mapping quality assessment from target data quality assessment (Fig. 5). Assessments that can be performed solely on the resulting FHIR representation, without considering the source data or transformation decision, were considered target data quality assessment. In contrast, assessments that require consideration of whether the target representation appropriately reflects the source data or transformation decision were considered source-to-target mapping quality assessment.

**Fig. 5.**
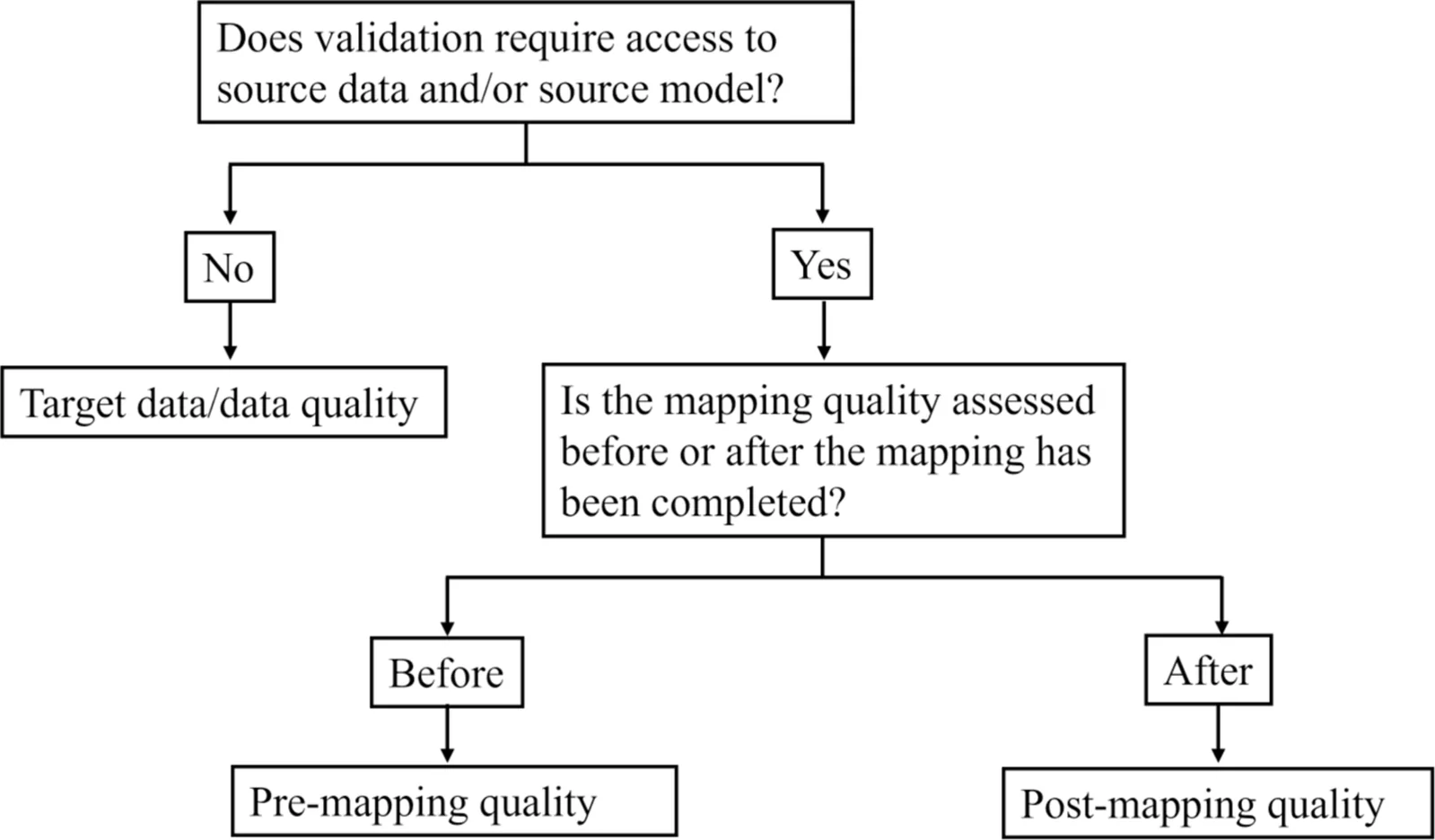
Decision model used to distinguish source-to-target mapping quality assessment from target data quality assessment. Target data quality assessment asks: “Is the resulting FHIR representation valid and well-formed?” In contrast, mapping-quality assessment asks: “Does the target representation accurately reflect the source data and the intended transformation decision?”. Manual and automated validation approaches may be applied at both stages.

In this study, data mapping was strictly defined as the transformation between source and target data models. Pipeline steps such as extraction (retrieving data without altering structure or semantics) and loading (writing data to the target system) were excluded, and related tools and challenges were not assessed.

### Literature search

The search was conducted across 3 bibliographic databases: IEEE Xplore, ACM Digital Library, and PubMed. In addition, a targeted search was performed within the JMIR journal family because of its strong focus on digital health, medical informatics, and health technology research. These databases were selected because together they cover a broad field relevant to FHIR data mapping, including biomedical informatics, healthcare interoperability, medical informatics, and computer science.

The same search string was used across all databases and the JMIR journal family: (“Fast Healthcare Interoperability Resources” OR FHIR) AND (mapping OR transform* OR convert* OR harmoniz* OR “data integration”) AND (“mapping quality” OR “mapping performance” OR “mapping accuracy” OR validation OR evaluation). The search strings were applied across all searchable fields available in each database, including titles, abstracts, keywords/indexing terms, and full text. No additional search filters were applied.

Each article identified through the search was evaluated for relevance and quality (screening) based on the eligibility criteria outlined in Table 2. The eligibility criteria were initially evaluated on a subset of articles (*n* = 20) to assess their feasibility. No modifications were required, and the criteria were deemed applicable. This process was performed in Rayyan (Rayyan Systems Inc., Qatar) by one reviewer (author M.S.).

**Table 2.** Eligibility criteria used in the literature search

Eligibility criteria
Inclusion criteria ◦ Peer-reviewed journal articles or conference papers written in English. ◦ Studies published up to and including 10 February 2026 ◦ Studies that describe, implement, or evaluate data mapping processes from a source dataset or data model to FHIR resources, with FHIR as the target representation. ◦ Studies addressing structural and/or semantic mapping, including aspects such as mapping coverage, representational fidelity, or transformation logic. Exlusion criteria ◦ Non–peer-reviewed publications (e.g., abstracts, editorials, commentaries, book reviews). ◦ Studies focused solely on FHIR data extraction, querying, or downstream analysis without describing or evaluating data mapping or transformation. ◦ Studies that do not explicitly report that FHIR mapping was performed, implemented, or evaluated.

After removal of duplicates (*n* = 20), a total of 742 unique articles were screened for title and abstract. After initial screening, 69 articles were included and further screened for full text (Fig. 6). After full text screening, 39 articles were finally included in the study. The data were extracted and charted using Microsoft Excel. The extracted data included the following data extraction categories: year of study, data to be mapped, mapping challenges, objective, FHIR resources used, mapping tools used, quality assessment methods (pre- or post-mapping, guided by Fig. 5) and quality assessment dimension used (if any), and associated mapping challenges. For data extraction and synthesizing, a descriptive thematic synthesis was conducted based on principles of thematic analysis described by Braun and Clarke [28]. The approach was adapted to focus on keyword-based coding and the identification of recurring patterns.

1. Full-text reading of included articles to identify information relevant to the research questions.
2. Organization of extracted information into logical groups and assignment of descriptive codes (interpretation in Table 3) corresponding to predefined data extraction categories.
3. Assignment of keywords to each coded data segment to capture common concepts (e.g., mapping tools, validation methods, and quality dimensions). Keywords referring to the same concept or tool (e.g., FML) were collated and grouped.
4. Synthesis of the grouped keywords and codes to identify recurring patterns across studies and to answer the research questions.

**Fig. 6.**
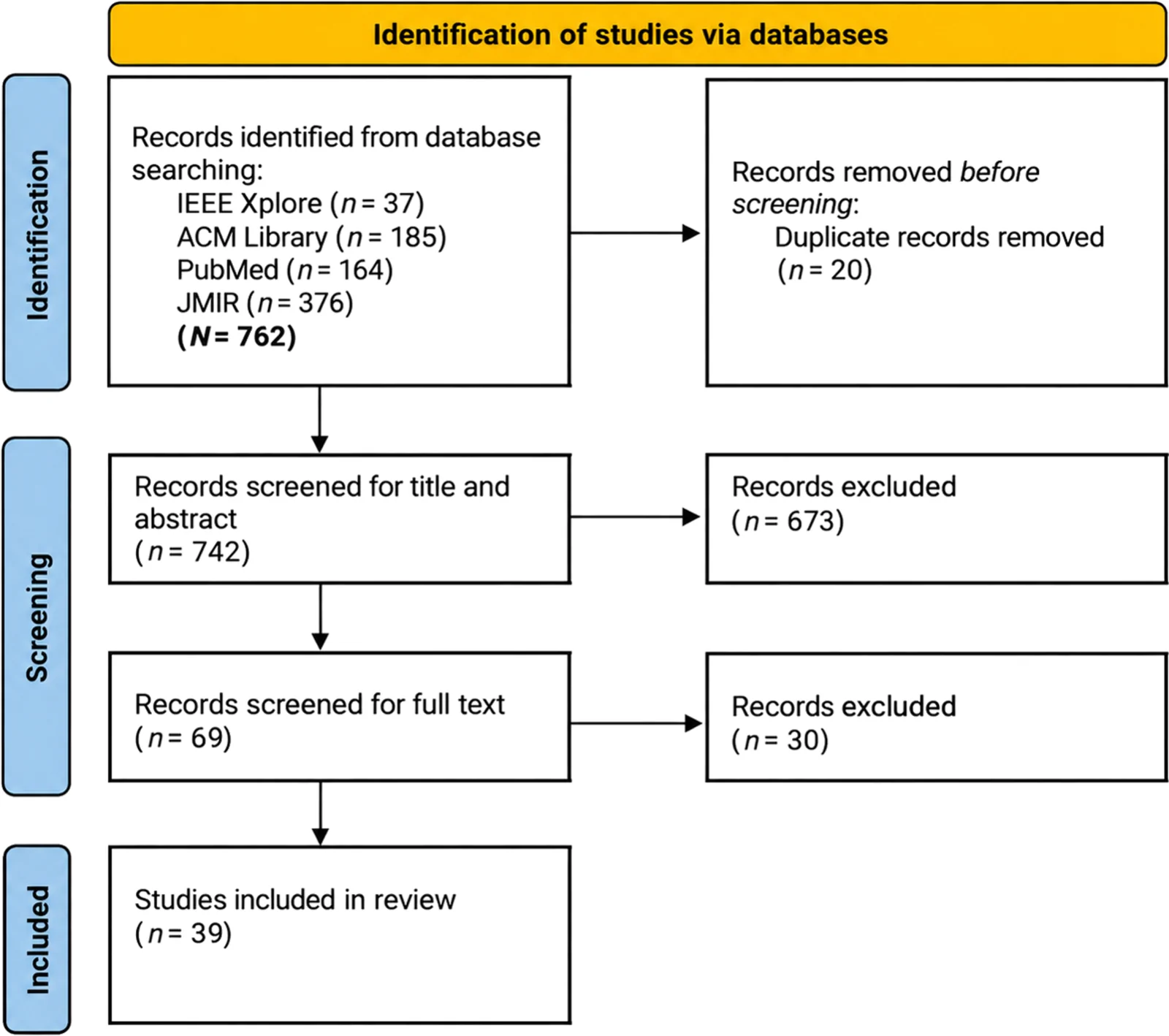
Study selection flowchart.

**Table 3.** Example of how the thematic analysis was conducted for 3 different data extraction categories: Mapping quality validation tool, mapping challenges, and mapping quality assessment dimension for article in [43]

Mapping quality validation tool ◦ Description in literature: “We validated the generated FHIR-based IPS against the IPS Bundle profile by means of the Inferno Validator … a web app for FHIR resource validation… In course of the validation, no errors were returned (only infos and warnings)… A corresponding validation could examine whether the mapped data within the IPS are complete and of practical value for clinicians” [43]. ◦ Interpretation: The resulting FHIR mapping was validated using Inferno Validator after completion of the mapping. ◦ Keywords: Mapping validation, Inferno Validator, post-mapping of target data representation only Mapping challenges ◦ Description in literature: “A limitation of the FML is that it does not allow to apply where-conditions on target instances. Depending on the content …, this can result in duplicate (same content but different UUIDs) resources to be generated, e.g. for specific organizations, within a Bundle” [43]. ◦ Interpretation: FML does not support “where-conditions”, meaning it can result in mapping duplicates in target data. ◦ Keywords: FML, duplicates, no where-conditions Mapping quality assessment dimension ◦ Description in literature: “Our results show that all required IPS sections can be supplied from ELGA data … Overview of which IPS sections can be populated from the defined source documents … The generated document successfully validates against the IPS profiles” [43]. ◦ Interpretation: Target data were evaluated for coverage and structural conformance through IPS section analysis and FHIR profile conformance validation ◦ Keywords: Coverage, structural conformance	

To improve consistency, coding was performed using predefined extraction categories (including mapping quality validation tool, mapping challenges, and mapping quality assessment dimension) and conceptual mapping quality dimensions. Codes were iteratively reviewed throughout the synthesis to ensure internally consistent application across studies.

To identify the gaps and to answer research question 2, all keywords for the mapping quality assessment dimension data extraction category were summarized. Each keyword was mapped by author M.S. to one of the dimensions listed in Table 1, based on either explicitly reported validation activities or explicitly described tool functionality intended to support the respective quality dimension. During the initial coding, each mapping was classified as either strong or weak, depending on the level of evidence supporting the assignment. A strong coding was assigned when the quality dimension was explicitly mentioned in the text or when 2 or more independent statements supported the same dimensions. Unclear mapping validation approaches and mappings supported by only a single indirect or implicit statement were classified as weak. All mappings initially classified as weak were subsequently re-screened by author M.S. to ensure consistency and reduce the risk of misclassification.

This synthesis enabled the identification of commonly reported quality assessment dimensions across studies, as well as gaps where specific dimensions were absent or underreported. In this study, no systematic forward or backward snowballing was performed. Findings from the literature search revealed commonly employed quality assessment dimensions and tools to assess FHIR mapping quality, as well as the challenges associated with them.

### Review-based refinement of conceptual dimensions and derivation of framework requirements

The conceptual contribution of this study was developed through the application and iterative refinement of the predefined mapping quality dimensions, synthesis of the scoping review findings, derivation of candidate framework requirements, and expert-informed refinement. The 7 mapping quality assessment dimensions defined in Table 1 provided the initial analytical framework for classifying the extracted mapping validation approaches, tools, reported quality assessment dimensions, and associated challenges. As described above, the definitions of these dimensions were refined iteratively when ambiguities became apparent during the synthesis, with all included studies subsequently revisited to ensure consistent application of the final definitions. The aim was not to validate a complete framework, but to identify and justify requirements that could guide future development of pre-mapping quality assessment approaches.

First, the extracted mapping validation approaches, tools, and associated challenges were analyzed and mapped to the 7 conceptual mapping quality dimensions defined in Table 1. This allowed identification of which dimensions were explicitly addressed in the literature, and which were underrepresented or absent. Figure 5 supports the identification of studies focusing on mapping quality rather than target data quality.

Second, the reported mapping challenges, such as structural mismatches, terminology inconsistencies, multiple plausible mapping targets, and reliance on custom profiles or extensions, were analyzed to identify recurring patterns that could influence mapping quality prior to transformation. These challenges were interpreted as indicators of potential mapping risks and complexity.

Third, gaps identified between commonly reported validation practices and observed mapping challenges were used to formulate and refine design requirements for a structured pre-mapping quality assessment framework. This process focused on identifying characteristics that could enable early detection of mapping ambiguity, structural incompatibility, terminology misalignment, and potential information loss.

This process followed an iterative and interpretive synthesis approach, in which the definitions and application of the predefined conceptual dimensions, as well as the derived framework requirements, were refined through repeated comparison with the extracted literature findings, identified mapping challenges, and established data quality frameworks. The resulting dimensions and requirements were subsequently reviewed and refined through structured consultation with 4 FHIR experts. Although the conceptual interpretation of the findings was discussed and refined through expert consultation, the experts did not independently validate the study selection or coding process.

### Expert consultation and requirement refinement

To strengthen the conceptual validity and practical relevance of the proposed mapping quality dimensions and framework requirements, a structured expert consultation was conducted with FHIR experts. Experts were identified through the FHIR Zulip community forum [29] by searching forum discussions containing the term “map”. Potential participants were identified based on demonstrated involvement in FHIR mapping discussions, including both individuals asking questions or reporting challenges related to mapping and individuals actively responding to mapping-related questions. In total, 25 experts were directly invited by email, and invited experts were also allowed to forward the invitation to relevant colleagues.

Experts were invited via email and provided with the full manuscript and a structured feedback form. The purpose of the consultation was clearly explained, and written consent was obtained from all participants prior to their involvement. Ethical approval was not required for this expert consultation and was therefore not obtained. The form included both quantitative ratings (5-point Likert scale [30]) and qualitative open-ended questions (see the Supplementary Materials). Experts were asked to evaluate:

• the relevance, clarity, and completeness of the proposed mapping quality dimensions.
• the relevance and feasibility of the proposed pre-mapping assessment framework requirements
• the conceptual distinction between data quality and mapping quality
• and the practical applicability of structured pre-mapping assessment in real-world FHIR implementations.

In addition, experts were asked to report on their professional background and experience with FHIR, as well as their perception of common mapping challenges, such as information loss, semantic distortion, and terminology mismatches. The manuscript and questionnaire were distributed to the selected FHIR experts by author M.S. via email. To enhance transparency while preserving participant anonymity, individual expert ratings and relevant qualitative feedback are reported in Tables S1 to S3 using anonymized expert identifiers (E1 to E4).

Responses were analyzed qualitatively to identify recurring themes and to assess conceptual clarity. Quantitative ratings were analyzed descriptively, with individual ratings used to illustrate variation in expert perspectives rather than to establish consensus. The expert feedback was incorporated into the Results and Discussion sections to refine definitions, clarify interpretations, and reflect considerations relevant to real-world FHIR implementation contexts.

## Results

The Results section presents the findings from the literature search (Table 4). A total of 762 articles were found, and after removal of duplicates, 742 articles were included in the literature search. Among these, 39 studies were included in the study (Fig. 6). These 39 included studies are listed in Table 4.

**Table 4.** Quality-related assessment activities reported across the 39 studies included in the scoping review. References listed in this table constitute the included study set used for the review synthesis. The “Validation/assessment approach” column lists the validation or assessment approaches explicitly reported in each study, whereas the “Quality assessment dimension addressed” column indicates the quality dimensions identified based on the authors’ reported methods, validation procedures, or tool functionality. Some of the tools listed under ‘Validation/assessment approach” column were not restricted to assessment of source-to-target mapping correctness. For example, validators such as Inferno Validator and HL7 FHIR Validator primarily assess properties of the resulting FHIR representation and were therefore classified according to the quality dimensions their reported functionality addressed. Their inclusion should not be interpreted as evidence that these tools assess source-to-target mapping correctness. “–” indicates that no specific validation approach could be identified from the published report.

Mapping approach and reference	Validation/assessment approach	Quality assessment dimension addressed	Data mapped
HAPI server, UML, manual mapping [4]	Manual comparison against SQL query results	Semantic correctness, retrievability, coverage	i2b2 clinical data model
FML [43]	FHIR Inferno Validator	Coverage, structural conformance, terminology accuracy	HL7 CDA
Manual mapping, UML, ISO/TS 20428:2017 [44]	Manual review	Coverage, semantic correctness, structural conformance	Molecular genomics (MolGen) report
Manual mapping [45]	Manual review, HAPI server	Coverage, retrievability	Genome sequencing (genomDE)
LINK-FHIR [46]	Gold standard dataset (MIMIC-IV on FHIR)	Terminology accuracy, semantic correctness, structural conformance	Medical Information Mart for Intensive Care IV (MIMIC-IV) dataset
FHIR ontology, [23]	Manual work for creating “Gold-standard”, Precision, Recall, F1	Semantic correctness	A vital signs dataset (heart rate) from a wearable stored in JSON
Manual mapping [47]	Manual review	Coverage, structural conformance	Microbiology findings, OpenEHR data first mapped to HL7 v2
TermX (FML and visual editor), manual [48]	Manual review, HAPI	Structural conformance, semantic correctness, completeness	HL7 CDA clinical documents
UMLS (terminology lookups), manual expert review [49]	Manual expert review	Completeness, semantic correctness, structural conformance	Spreadsheet data about family planning and sexually transmitted infections
vcf2fhir (open-source Python program) [50]	Manual review through HL7 FHIR connectathon/integration testing events	Structural conformance, coverage	Variant Call Format (VCF) containing genomics data
FML (FML2Mirth), manually, Mirth Connect ( open-source server) [19]	HAPI Validator, JUnit testing framework	Structural conformance, terminology accuracy	20 anonymized laboratory data
ETL pipeline (FML based) [37]	Manual review	Coverage, completeness, terminology accuracy, structural conformance	Oncology dataset in CSV
Manual mapping [51]	HL7 Validator, manual expert review	Coverage, semantic correctness, terminology accuracy, structural conformance	COVID-19 research data
FML [14]	FHIRPath queries on HAPI server, Python scripts, manual review, Matchbox FHIR templating engine	Coverage, completeness, semantic correctness, structural conformance, terminology accuracy	CSV dataset on unilateral trans-femoral amputations
Open-source (dicom-fhir-converter) [52]	Manual inspection based on output from Pipeline	Coverage, terminology accuracy, structural conformance	DICOM imaging metadata
Manual mapping [53]	Manual review, Boolean scoring system	Coverage, structural conformance	Ophthalmology dataset
Manual mapping [54]	Manual review	Coverage, inter-rater reliability	Cardiovascular dataset
Manual mapping, NLM Form Builder [55]	Manual review	Coverage, completeness, terminology accuracy	Pathology cancer reporting protocols (check-sheets)
Manual mapping, NLP2FHIR [56]	Manual review	Terminology accuracy	Oncology genetic reports
Ontology Mapper [57]	Manual review	Semantic correctness	Healthcare dataset in CSV (citizen data)
Manual mapping [6]	Manual review	Coverage, semantic correctness, terminology accuracy	Self-reported records about the genetic test
Manual mapping [58]	–	Coverage	Diabetes mellitus Dataset
Manual mapping [38]	HAPI server, SIMPLIFIER.NET	Coverage, completeness, structural conformance,	Emergency medical teams records
Ontology-based manual mapping [59]	Multi-layer harmonization analysis (including manual expert review)	Coverage, completeness, structural conformance, terminology accuracy	European Rare Disease Registry
Custom software [60]	Custom query tool	Retrievability	EHR data from several hospital databases
Manual mapping [61]	Manual review	Coverage, completeness, semantic correctness, inter-rater reliability	Registry data elements from public data dictionaries
FAIR4Health Data Curation Tool (open-source ETL-based mapping tool) [62]	Manual review	Structural conformance	Legacy health research data from FAIR4Health pilot sites, stored in relational databases, Excel and CSV
CAMP FHIR (custom, open-source Clinical Asset Mapping Program for FHIR) [63]	Manual review	Semantic correctness, coverage	Clinical data from i2b2
Manual mapping [64]	Manual review, forge	Coverage, structural conformance, semantic correctness	Structured clinical genomic sequencing report data (ISO/TS 20428)
Manual mapping HAPI server, [65]	Manual review	Coverage, inter-rater reliability	Clinical trial registry metadata
ETL-based mapping approach [66]	Manual review	Coverage, structural conformance, completeness	Demographic, diagnostic, and treatment data for childhood cancer survivors
Manual mapping [67]	Manual review	Coverage	Paper-based emergency medical services (EMS) patient care report data
Manual mapping, ETL pipeline [68]	-	Coverage	Adult inpatient EHR data
Manual mapping [69]	Manual review	Coverage	Electronic health records
NLP2FHIR [70]	Manual review, gold standard labels	Semantic correctness	Semi-/unstructured discharge summaries
Manual mapping, proprietary mapping language [36]	Manual review	Coverage, completeness, structural conformance, semantic correctness, terminology accuracy	Health datasets from hospital EHRs and research registries in CSV or Excel
Manual mapping [24]	Manual review	Coverage, inter-rater reliability	Electronic case report forms from 3 multi-site clinical research studies
Manual mapping [71]	Manual review, custom tool	Coverage	Pediatric studies’ case report forms
Ontology alignment, ETL pipeline [10]	Manual review mapping refinement, ground-truth definition (Precision/Recall/F1)	Semantic correctness, terminology accuracy, completeness, structural conformance	Data related to Medicare claims and patient demographic

i2b2, informatics for integrating biology and the bedside; FML, FHIR mapping language; UML, unified modeling language; UMLS, unified medical language system; LLM, large language models; NLM, National Library of Medicine; LINK-FHIR, LLM-integrated normalization and knowledge for FHIR; DICOM, digital imaging and communications in medicine; ETL, extract transform load

### Current mapping quality validation tools and quality assessment dimension

All mapping quality assessment activities identified across the 39 included studies could be mapped to at least one of the quality dimensions defined in Table 1. Previous mapping efforts described in the literature can be found in Table 4. The distribution of the 7 mapping quality assessment dimensions across individual studies is further illustrated in Fig. S1, highlighting differences in the number and combination of dimensions addressed across the included studies.

The frequencies reported below represent quality assessment dimensions addressed during FHIR mapping evaluation and may include both source-to-target mapping assessment and assessment of properties of the resulting FHIR representation. According to the studies summarized in Table 4 (*n* = 39), mapping was predominantly performed using manual mapping approaches or manual inspection workflows (24/39, 62%). Rule-based mapping using FML was the most frequently reported explicit mapping language (5/39, 13%). Manual expert review/manual review was the dominant validation approach (31/39, 80%), whereas automated validators (e.g., HAPI/Inferno/HL7 validator) were reported in a minority of studies (6/39, 15%) and were most often applied as post-mapping target conformance checks rather than source-to-target validation. In terms of the 7 quality assessment dimensions, coverage was the most commonly identified dimension (28/39, 72%), followed by structural conformance (20/39, 51%) and semantic correctness (16/39, 41%). Terminology accuracy (13/39, 33%) and completeness (11/39, 28%) were identified less frequently, while inter-rater reliability (4/39, 10%) and retrievability (3/39, 8%) were rarely addressed. Overall, the findings show that mapping quality assessment remains fragmented, with considerable variation in the dimensions addressed across studies.

### Mapping challenges

Table 5 summarizes reported challenges with FHIR data mapping and mapping quality assessment identified in the included studies. Only studies where challenges could be extracted using the thematic analysis approach are included in Table 5.

**Table 5.** Mapping challenges and challenges associated with the quality assessment validation. Each resource in the Ref column can be found in Table 4. Importantly, some of the identified challenges were not conceptualized as independent mapping quality dimensions because they represent implementation and governance considerations that influence multiple quality dimensions simultaneously. Instead, the challenges presented in this table informed the design requirements of the proposed framework.

Ref	Mapping challenges	Quality assessment challenges
[71]	One-to-one mapping rarely exists, requires transformation logic (split/merge fields), mapping ambiguities, human/manual review requires supervision, dependent on annotation quality	Precision/Recall requires a “Ground-truth”, quality metric reliability depends on expert labeling, dependent on annotation quality
[4]	Structural mismatches (row-based data *vs*. hierarchical resources), FHIR often need profiling (difficult to determine what constraints, fields, etc.), knowledge-intensive and ambiguous, limited FHIR coverage	Manual and labor-intensive, evaluation mostly proves output equivalence of target data, not validation of source-to-target
[43]	Requires local and custom solutions often based on manual mapping work, FML can result in duplicates	Validation outcomes are tool-dependent, lack of clear interpretation of mapping results
[44]	Legacy/unstructured storage of high dimensionality data (PDF reports, local formats) increase mapping burden, limited coverage (over-reliance of extensions), heterogeneity in source data force the use of profiles/extensions, mapping can be blocked by missing codes/terminology content	Structural limits and mismatches (FHIR required fields not part of source dataset), quality dependent on implementer interpretability
[45]	Limited FHIR coverage, incompatibility between different FHIR IG versions, missing fields in source dataset requires transformation logic (calculate age), over-reliance of extensions/profiling	Need certain specialized tools to perform the quality assessment (compatible FHIR servers, etc.)
[46]	Structural differences between source and aimed target dataset, NLP approaches require extensive labeled training data, tools dependent	NLP tools depend heavily on the gold standard’s scope/assumptions, subtle character differences (e.g., ICD-10), yield big semantic errors
[23]	Heterogeneous formats, structural differences between source and target data, ambiguous semantic matches (multiple plausible FHIR targets)	Quality assessment depends on having gold/reference alignment, difficult to know when mapping is “good enough”
[47]	Structural differences between source and target data (target model is less expressive), version mismatches	Information loss
[48]	Mapping ambiguous and context-dependent (no single correct way), structural differences between source and target dataset, version-sensitive, lack of universal mapping approach	Lack of tools for quality assessment, dependent on domain expert review and manual inspection, information loss
[49]	Mapping ambiguities (no-single correct way), context dependent, structural differences between source and target dataset, difficult to determine use of profiles (reuse, extend, custom)	Information loss
[19]	Structural differences between source and target data (source/target model is less expressive, information incompleteness	Information loss, quality dependent on mapping tool used
[51]	No single truth, not always one-to-one mappings, difficult to align with terminology codes	Manual review revealed ambiguities and nonmatching mappings that required iterative consensus negotiation
[14]	Heterogeneous/proprietary clinical data formats, dependent on source data quality, (data preprocessing), structural differences between source and target data (tabular data *vs*. hierarchical structure), custom terminology codes	Tool dependent, manual work creates inconsistencies and ambiguities
[53]	Coverage gaps, manual interpretations create ambiguities	Use-case dependent
[54]	Manual work due to lack of tools, heavy reliance on extensions due to poor coverage	Quality assessment is manual, expert-dependent and hard to reproduce, lack of tools
[69]	Heterogeneous/proprietary source dataset, difficult to find corresponding elements	Information loss
[55]	Structural differences between source and target data (nested data, free-text fields), unclear meaning of source data	Preserve semantic meaning, not one-to-one mappings, version mismatch of source and target data
[57]	Heterogeneous/proprietary source dataset makes transformation hard, contradicting/overlapping relationships and terminologies, several intermediate steps increase mapping complexity	Need for reference standard
[38]	Messy source data structure (paper based and Excel sheets), ambiguities associated with manual work, coverage gaps, semantic mismatches	Poor coverage per domain, limited coverage of current tools (focusing mostly on profile conformance of target dataset)
[59]	Direct mapping was not always possible nor one-to-one, elements had to be excluded, structural mismatches between source and target dataset (data type mismatch), mapping quality depends heavily on input data quality	Manual work creates heavy workloads
[60]	Dependent on source data quality (high dimensionality, heterogeneity, noise, sparseness), redundant and inconsistent terminologies in source data, use of custom software for data transformation	Information loss, source dataset characteristics influence mapping quality
[61]	Poor FHIR coverage, poor source data quality, terminology mismatches and binding strengths constraints, no clear one-to-one mapping (several possible mappings exist for the same field), source dataset spans multiple FHIR resources	Subjectivity and inter-annotator disagreement associated with manual work, information loss due to no direct matches mismatch in granularity
[62]	Structural differences between source and target data (mismatch between available fields and required fields), proprietary terminology codes, difficult to find code via URI	Insufficient detail about validation results (hard to trust mappings), poor quality transparency
[63]	Coverage gaps, difficult to find exact matches, mapping requires various intermediate steps	Information loss, change of data meaning, loss of granularity
[64]	Difficult to find FHIR equivalent, overuse of extensions, lack of standardized vocabulary	Lack of formal quality assessment framework, extensions, and proliferation limits validity
[68]	Limited FHIR coverage, lack of IG (need for custom IG)	No formal evaluation of mapping approach
[24]	Limited FHIR coverage, data need to be spread across several FHIR resources, mapping requires several intermediate steps (i.e., transformation to an intermediate format)	Mapping coverage does not mean correctness of mapping, tools dependent

IG, implementation guide; URI, uniform resource identifier

Overall, the findings in Table 5 show that mapping challenges are rarely caused by a single factor. Instead, they emerge from (a) the interaction between structural mismatches between source representations and FHIR’s hierarchical resource model, (b) semantic ambiguity where multiple FHIR targets are plausible and there is no single “correct” mapping, and (c) coverage and terminology limitations, including missing fields in the source dataset, incomplete terminology bindings, and frequent reliance on custom profiles, leading to “profile proliferation”—the rapid growth of use-case-specific profiles that results in overlapping or incompatible artifacts [31].

Taken together, FHIR mapping depends on local context (domain and source dataset), tools used, and interpretive decisions, while mapping quality assessment lacks standardized, scalable methods that can be applied early and consistently across settings. This motivates the need for approaches that (a) reduce ambiguity during mapping, (b) detect predictable mapping risks (such as information loss, proliferation, or reliance on poorly governed or project-specific extensions) before transformation begins, and (c) complement to post-mapping conformance checks, with stronger emphasis on source-to-target correctness.

### Mapping gaps and design requirements for pre-mapping quality assessment tools

Current literature reveals that FHIR mapping quality assessment remains fragmented, with many approaches focusing on assessment of the resulting FHIR representation rather than the source-to-target mapping decision itself. Coverage was the most frequently identified quality dimension, followed by structural conformance and semantic correctness (Table 4). Additionally, in comparison to how data quality (target dataset only) is evaluated using several quality assessments dimensions, assessing data mapping quality (source-target dataset) seems to lack a corresponding approach.

The challenges presented in Table 5 indicate that conventional post-mapping checks are insufficient. To address the gaps and challenges presented in Table 5, such as limited FHIR coverage, no clear one-to-one mappings, over-reliance of extensions, structural differences between source and target dataset, multiple plausible mappings (no-single correct way), and lacking source data quality, a complementary tool is required. Based on findings from the thematic synthesis, an initial set of candidate requirements was formulated by the first author (M.S.). These requirements were subsequently refined through structured consultation with 4 FHIR domain experts to improve clarity, relevance, and practical applicability: (a) Pre-mapping focus: Enables validation of data structures and mappings on source and target dataset, before starting the mapping process, allowing potential issues to be addressed early in the process. (b) Accessible to multidisciplinary users: Designed to reduce unnecessary technical barriers and support use by multidisciplinary stakeholders while recognizing that appropriate FHIR, terminology, and domain expertise may still be required for mapping decisions. (c) Interpretable and actionable: Presents validation results in a clear and interpretable manner that supports rapid understanding and decision-making that informs users of potential risks, such as heavy reliance on custom extensions, data type transformations, or information and semantic loss. (d) FHIR-native: Builds upon established FHIR best practices, including appropriate selection of FHIR resources, profiles, extensions, terminology bindings, and consideration of FHIR maturity model (FMM) levels, profile governance, and adoption. The assessment should consider alignment with established and widely adopted FHIR implementation guides, profiles, and extensions rather than assuming that reliance on Core FHIR resources alone represents higher mapping quality. (e) Comprehensive quality assessment: Evaluates the most critical aspects of mapping quality in alignment with Table 1 and FHIR’s design principles (i.e., extensions, terminology binding strength, and maturity levels according to FMM). (f) Context-adaptable: Supports adaptation to different use cases and datasets, including customization of quality assessment dimensions to account for domain-specific variations in FHIR coverage.

These requirements could guide the development of a future framework, helping reviewers to answer questions such as: Is this source dataset suitable for FHIR transformation? Did we represent the data correctly and meaningfully in FHIR? What resources should we use? Is the intended mapping straightforward, or is it open to multiple interpretations? Would different implementers or teams likely produce different mappings for the same data element? Does FHIR adequately cover the source data element using established resources, profiles, or extensions, and are any required custom artifacts justified? What are the risks associated with mapping?

To illustrate how the proposed requirements could support FHIR mapping work in practice, we return to the OSISP case introduced in the Introduction. This example is not intended to implement a complete framework, but to demonstrate how pre-mapping assessment could help identify ambiguity, semantic divergence, and potential interoperability risks before transformation rules are defined. Furthermore, the provided example should be viewed as a candidate representation illustrating one plausible mapping alternative, rather than as a recommendation. Appropriate representations may depend on the applicable FHIR version, implementation guides, profiles, jurisdictional requirements, and intended use.

### Illustrative example of pre-mapping risk identification

The example in this section builds on the Care@Distance team’s attempt to map data from trauma AI model to FHIR [16]. As described earlier, the source data included AI model inputs and outputs, intended use, validation information, bias-related information, and algorithm type. Several of these elements did not have obvious one-to-one representations in FHIR, making them useful for illustrating why pre-mapping quality assessment may be needed.

Consider the source data element “intended use”, defined as: “identify severely injured trauma patients in the prehospital setting”. Before any transformation is performed, this element can already be interpreted in different ways. Reviewer A may interpret it as describing the target population and clinical outcome, and therefore map it using EvidenceVariable together with DeviceDefinition. Reviewer B may instead interpret it as describing the regulatory purpose of a Software as a Medical Device, and therefore represent it using ArtifactAssessment and a custom extension on DeviceDefinition.

Both representations may pass post-mapping validation, since the generated FHIR resources can be structurally valid and conformant to relevant profiles. However, they encode different semantic interpretations of the same source element. This illustrates a key limitation of post-mapping validation: It can confirm that the target representation is technically valid, but it may not reveal whether the mapping decision was semantically appropriate, reproducible, or aligned with the intended use of the source data.

This example shows how mapping risks can arise before transformation begins. A structured pre-mapping assessment could make such risks explicit by identifying multiple plausible target resources, reliance on custom extensions or profiles, terminology uncertainty, and possible loss or distortion of meaning. In this way, the framework would support more transparent and reproducible mapping decisions before formal mapping rules are implemented. Similar ambiguity may arise when mapping additional elements such as response time (<8, ≥8, unknown), dominating type of injury (blunt, penetrating, unknown), and algorithm task type (supervised, unsupervised, semi-supervised, reinforcement learning) [16] (Table 6).

**Table 6.** An example showcasing how a framework could reveal source data ambiguities and uncertainties to highlight potential fragmentation risks, information loss, and implementation complexities

Field	FHIR fields (examples)	Primary risks
Response time	Encounter.period/Observation.valueQuantity/derived category	Structural ambiguity; multiple plausible targets; retrievability impact
Dominating injury	Condition.code/Observation.code	Semantic ambiguity; terminology binding risk
Algorithm task type	PlanDefinition/Library/DeviceDefinition + extension	Limited core coverage; extension dependence; governance burden

### Findings from structured FHIR expert consultation (*n* = 4)

#### Expert characteristics and experience

Six (6/25) experts responded to the consultation, of whom 4 provided sufficiently detailed additional feedback that could be included in the qualitative analysis. Four independent FHIR experts from different European contexts, including the Nordic countries, with experience ranging from participation in HL7 communities to practical FHIR mapping work within the public sector, participated in the structured consultation. Participants had professional experience as FHIR implementers, system or solution architects, consultants, and contributors to HL7 activities. Reported experience working with FHIR ranged from 2 to 5 years to more than 10 years, and all experts reported prior experience both mapping data to FHIR and reviewing mappings created by others. This provided perspectives from individuals with practical experience in FHIR implementation and interoperability projects.

#### Perceived frequency of mapping challenges in real-world implementations

The individual expert ratings are presented in Table 7. Given the small number of participants (*n* = 4), the ratings were interpreted descriptively rather than as evidence of expert consensus. Terminology mismatches were generally rated as occurring more frequently (ratings 3 to 5), whereas information loss (ratings 1 to 2) and semantic distortion (all ratings 2) were rated as less frequent. These ratings suggest that terminology mismatches were perceived as more frequent than information loss or semantic distortion among the participating experts.

**Table 7.** Expert ratings of mapping challenges (*n* = 4). Scale: 1 = rarely, 2 = occasionally, 3 = sometimes, 4 = frequently, 5 = very frequently.

Challenge	Expert 1	Expert 2	Expert 3	Expert 4
Information loss	1	2	1	1
Semantic distortion	2	2	2	2
Overreliance on custom extensions	3	3	3	3
Overreliance on custom profiles	2	2	N/A	2
Ambiguity between plausible FHIR resources	3	4	2	2
Terminology mismatches	3	4	5	3

N/A, not rated

#### Expert evaluation of proposed mapping quality dimensions

Experts generally considered the proposed mapping quality dimensions to be relevant and conceptually meaningful. Coverage, semantic correctness, terminology accuracy, and structural conformance generally received high relevance ratings (Table S1). However, individual ratings varied across dimensions, reflecting differences in how their relevance was perceived across implementation contexts. Respondents also noted several important nuances. First, coverage does not necessarily imply practical value, as full representation of the source dataset may not always be required depending on the intended use case. Second, semantic correctness can be difficult to define objectively, since multiple modeling approaches may be valid within the flexible FHIR resource model. Third, terminology accuracy was described as strongly influenced by project-specific profiles, terminology governance, and jurisdictional standards rather than by the base FHIR specification alone. Finally, experts observed that structural conformance and completeness may overlap with validation tasks already supported by existing FHIR validator tools, suggesting that these dimensions may partially reflect data quality validation rather than mapping-specific evaluation.

Some experts also proposed additional considerations that may influence mapping quality, including FHIR resource lifecycle maturity and the potential evolution of resources across future FHIR releases.

Overall, the expert feedback indicates that while the proposed dimensions are broadly applicable, their relative importance may vary depending on the implementation context, domain requirements, and governance environment.

#### Expert evaluation of framework requirements

Experts generally considered the proposed design requirements conceptually relevant, although responses regarding feasibility and practical necessity varied.

Individual ratings of the proposed framework requirements are presented in Table S3. Ratings of the pre-mapping focus requirement varied across experts (2 to 5), although 3 of the 4 experts rated its relevance as high or essential (4 to 5). Ratings also varied across the remaining requirements, reflecting differences in perceived relevance and feasibility across implementation contexts (Table S3). Requirements related to accessible to multidisciplinary users and interpretable and actionable output were rated positively, particularly for facilitating communication with clinical stakeholders and multidisciplinary project teams.

Overall, experts noted that mapping evaluation typically occurs implicitly during design discussions and suggested that a more structured and transparent assessment approach could improve both quality and explainability of mapping decisions. Furthermore, the responses indicate that a structured pre-mapping assessment approach could improve transparency, explainability, and consensus-building during mapping design activities.

#### General feedback on the applicability of the proposed requirements

Multiple experts emphasized that mapping quality cannot be evaluated independently of the FHIR profiles and FHIR implementation guides used in a given project, as these define domain-specific constraints, terminology bindings, and structural refinements that influence how source data can be represented. Also, experts noted that the use of custom profiles and extensions does not inherently indicate poor mapping quality but rather reflects limitations in core resource coverage or domain-specific requirements. Instead, mapping quality should be assessed based on how well the selected representation preserves semantic intent, supports interoperability, and aligns with established domain conventions. Finally, several experts emphasized the importance of alignment with existing standards, implementation guides, and jurisdictional models, as these often influence mapping decisions in real-world implementations.

#### Impact of expert feedback on framework refinement

Expert feedback contributed to refinement of the proposed mapping quality dimensions and framework requirements, particularly by clarifying the role of profiles, extensions, terminology bindings, and implementation context in mapping quality assessment.

Experts suggested grouping the 7 quality dimensions into syntactic quality (structural conformance, completeness, and coverage) and semantic quality (semantic correctness, terminology accuracy, retrievability, and inter-rater reliability). However, this grouping was not adopted in the final framework. Although the distinction may be useful for some dimensions, retrievability primarily concerns downstream usability, while inter-rater reliability concerns reproducibility of the mapping process, and neither can be unambiguously classified as a purely semantic property. The 7 dimensions were therefore retained as separate quality assessment dimensions.

Expert feedback also highlighted that the use of custom profiles and extensions does not inherently indicate poor mapping quality. Rather, their appropriateness depends on the implementation context, available FHIR resources, relevant implementation guides, and domain-specific requirements. Accordingly, the FHIR-native requirement (requirement 4) was refined to emphasize alignment with established and widely adopted FHIR resources, profiles, extensions, terminology bindings, implementation guides, maturity levels, and governance considerations, rather than prioritizing Core FHIR resources alone.

## Discussion

### Principal findings and relation to previous work

This study extends previous research on FHIR mapping work by shifting the focus from mapping techniques to mapping quality assessment work. While much of the existing literature focuses on mapping techniques or the use of interoperable health data [14,32–35], this review focuses on the processes that enable interoperable data to be created in the first place. Rather than asking “How can FHIR data mapping be performed more effectively?” or “how can FHIR data be used to achieve interoperability”, this review asks, “How can the quality of FHIR data mapping be systematically assessed to ensure that mapping decisions are reproducible, valid, and accurate?”. By identifying how mapping quality is currently assessed and where important gaps remain, this study seeks to support the development of more reliable and reproducible source-to-target FHIR-data mapping tools.

The results of this review include 4 main findings. First, the assessment of FHIR mapping quality remains fragmented, predominantly manual, with many of the identified approaches assessing the resulting FHIR representation rather than the source-to-target mapping decision itself (Fig. 4 and Table 5). This is also reflected by structural conformance being one of the most frequently identified quality assessment dimensions (20/39, 51%), which partly reflects an emphasis on the quality of the resulting FHIR representation rather than on source-to-target mapping correctness.

Second, many mapping tools seem to rely on predefined rules [20–22], with a lack of transparency in how these rules are established [8]. Interestingly, despite frequent reports of mapping ambiguity and dependence on manual interpretation, inter-rater reliability was identified in only 4/39 studies (10%), suggesting that reproducibility between independent mappers remains comparatively underexamined. This pattern may partly reflect how FHIR mapping work is commonly conducted. Many studies describe mappings performed by a single implementer or developed collaboratively, or iteratively, through expert discussion. In such settings, agreement may be reached through consensus without formally quantifying whether independent mappers would arrive at the same representation. However, in healthcare contexts, where local data structures, clinical context, and implementation requirements often shape mapping decisions [22,32,36], we believe that formal assessment of agreement between independent mappers may be particularly relevant.

Third, the frequency with which the different quality dimensions were identified varied considerably across the included studies (Table 4). Existing validation approaches most commonly assessed whether the source data could be adequately represented in FHIR (coverage assessed in 28/39, 72% studies), or if the resulting FHIR representation was structurally valid (structural conformance assessed in 20/39, 51% studies), whereas other dimensions, such as retrievability, were rarely evaluated (3/39, 8%). For example, structural conformance can be assessed independently of the intended downstream use using established FHIR validation tools. Retrievability, in contrast, may become relevant primarily when the resulting FHIR data are intended to support retrieval or RESTful application workflows. The low frequency of retrievability assessment may therefore reflect the scope and intended use of the included mapping studies rather than indicating that this dimension is considered unimportant or that tools for its assessment are unavailable.

Lastly, the included studies lacked a standardized approach for evaluating mapping quality across different quality dimensions. To facilitate the thematic analysis, there was a need to operationalize the different aspects of quality assessment reported in the context of FHIR mapping. The 7 quality assessment dimensions identified in this review should consequently be understood as an analytical framework for organizing the heterogeneous assessment activities reported across the included studies, rather than as 7 equivalent or independent properties of source-to-target mapping quality. This framework allowed the heterogeneous mapping quality work reported in the included studies to be systematically analyzed and grouped. Together with the identified gaps and challenges, the findings informed the formulation of 6 expert-informed requirements for future pre-mapping assessment approaches. These contributions introduce a pre-mapping perspective, where mapping decisions, ambiguities, and potential quality risks can be considered before transformation rules are implemented, complementing existing approaches that assess mappings or the resulting FHIR representation after transformation.

We believe that this pre-mapping perspective is important because multiple target representations may be structurally valid while representing different interpretations of the same source data. This raises a fundamental question: Is mapping quality simply data quality as perceived by the receiving system? If the receiving system can process the mapped data appropriately, does it really matter how the mapping was performed? We argue that it does. Although one receiving system may successfully process a particular mapping, this does not guarantee that another system, another implementation, or a future use case will retrieve, interpret, and use the data in the same way. Mapping quality is therefore not only about enabling a single successful data exchange but about preserving the intended meaning of the source data in a way that supports consistent interpretation across systems and over time. In this sense, we view mapping quality as a prerequisite for avoiding ad hoc mapping solutions that undermine semantic interoperability and reproducibility. Hence, the result from this review suggests that mapping outcomes still vary depending on implementation choices and local needs, highlighting the need for more transparent, reproducible, and standardized approaches to data mapping validation.

### Reliability and interpretation of the thematic synthesis

The thematic synthesis and quality assessment were performed by a single reviewer (M.S.), and no formal independent double-coding or inter-rater agreement analysis was conducted. Consequently, the findings in this review, particular in Tables 4 and 5, are potentially subject to reviewer interpretation, and different reviewers may have classified some studies differently.

This limitation was particularly relevant because mapping validation was not always reported as a distinct activity performed after the mapping process. Instead, activities related to mapping quality were sometimes incorporated into the mapping process itself or could only be identified indirectly from descriptions of how the mapping was performed. For example, one study [37] stated that “The results demonstrate a perfect one-to-one mapping across all stages...” Although this was not explicitly described as a post-mapping validation activity, the statement indicates that the authors assessed whether the source dataset could be fully represented in the FHIR target data and was therefore considered evidence of mapping quality assessment. Similar ambiguity occurred when attempting to distinguish validation of the source-to-target mapping from validation of the resulting FHIR representation. Studies describing mapping tools, such as FHIR Inferno Validator, introduced an additional challenge, as tool functionality intended to support a specific quality dimension (such as structural conformance for Inferno) could be reported without a formal evaluation of the resulting mappings.

To reduce the influence of subjective interpretation, the thematic analysis was conducted using predefined and operationalized quality dimensions, with explicit keywords and criteria used to guide classification. Evidence was initially distinguished according to the strength of support, with strong evidence requiring explicit reporting or multiple supporting statements, while weaker or indirect evidence was subjected to additional screening by author M.S. This approach does not eliminate the risks associated with a single-reviewer approach but provides a more systematic basis for enhanced transparency and reproducibility.

Accordingly, the results should be interpreted as an indication of overall patterns rather than as an absolute classification of each individual study. This means that the reported frequencies of individual quality dimensions should not be interpreted as exact measurements of how often a particular aspect of mapping quality is considered in practice. Rather, the results presented in this review provide an indication of how often sufficient evidence for each dimension could be identified in the published studies using the operational definitions applied in this review. In ambiguous cases, different definitions or independent reviewers could reasonably have reached different classifications. Nevertheless, we consider such differences unlikely to alter the main conclusions of the review, as the broader patterns, including heterogeneity in validation approaches, uneven coverage of quality dimensions, and the absence of a standardized approach to mapping quality assessment, were consistently observed across the included studies and in previous practical implementation work.

### FHIR could benefit from pre-mapping quality validation

The illustrative examples involving the OSISP algorithm in this study (III.D Illustrative Example of Pre-Mapping Risk Identification) demonstrated how multiple structurally valid mappings may represent different semantic interpretations of the same source data, reinforcing the limitations of target-only validation approaches. These observations are consistent with findings from the expert consultation and our own practical experiences, suggesting that several mapping challenges could potentially be mitigated through structured pre-mapping quality assessment. Prior studies have similarly highlighted the value of identifying mapping issues early in the process [38,39], including reducing reliance on workarounds (e.g., redundant extensions or local terminologies) [38], and improving the early detection and mitigation of integration failures and information loss [39]. This may be particularly relevant for datasets containing elements not fully represented in existing standards, which are common in FHIR integration and often addressed through extensions or profiling.

For example, Garza *et al*. [24] reported that FHIR coverage ranged between 45% and 79% depending on the study type and focus area. In other cases, excessive reliance on FHIR extensions may introduce additional challenges related to data query or search [17,40]. Overall, these findings indicate that FHIR coverage is highly dependent on domain characteristics and data granularity [15,41,42], which further reinforces the need for a more systematic and reproducible mapping approach.

### Future framework development and relationship to existing FHIR tools

The findings of this study suggest that although FHIR has become a dominant framework for healthcare interoperability, mapping data to FHIR remains a substantial practical and conceptual challenge [20]. The review findings and expert consultation indicate the need for more structured approaches to pre-mapping quality assessment that can complement existing post-mapping validation tools, such as the HL7 FHIR Validator, HAPI, and IG Publisher. With this said, the idea for a new FHIR mapping validation tool should not be to replace existing tools, but rather to address the gaps presented in this review.

Rather than proposing a fully validated framework, this study identifies conceptual requirements that could guide future framework development. Such approaches should focus on identifying ambiguities, structural mismatches, terminology inconsistencies, and other characteristics that may increase mapping complexity or introduce risks related to semantic distortion, information loss, or reduced interoperability. A future pre-mapping assessment framework could therefore support more transparent, explainable, and reproducible mapping decisions before transformation rules are implemented.

## Summary and Outlook

This review identified a fragmented landscape of FHIR mapping quality assessment characterized by substantial reliance on manual review, heterogeneous validation practices, and limited attention to source-to-target correctness. To address these gaps, 7 conceptual mapping quality dimensions and 6 preliminary requirements for future pre-mapping assessment approaches were proposed and refined through expert consultation.

The findings suggest several directions for future work. First, the proposed dimensions and requirements should be further refined together with the HL7 FHIR community and translated into an operational framework and evaluated across multiple FHIR implementation contexts and domains. Such evaluation should investigate whether structured pre-mapping assessments can improve consistency, reduce ambiguity, and increase reproducibility between independent implementers. Since mapping quality appears highly dependent on local implementation choices, future frameworks may also need mechanisms for incorporating contextual information such as implementation guides, profile maturity, and domain-specific constraints.

Finally, emerging AI-based and large language model (LLM)-assisted mapping approaches represent a promising area for future investigation. As automated mapping techniques become increasingly common, transparent approaches for assessing mapping assumptions, explainability, and semantic correctness before transformation may become increasingly important.

## Limitations

Several assumptions, definitions, and interpretations may have influenced this review. Findings in Tables 4 and 5 rely on a single reviewer’s (M.S.) FHIR expertise and subjective assessments, including the use of custom mapping quality dimensions (Table 1). While this enabled analysis, no independent double-coding or inter-rater agreement assessment was conducted, meaning that different reviewers may have produced alternative definitions. Furthermore, no widely adopted framework for evaluating FHIR mapping quality exists, which limits comparability across studies and motivates the development of our own mapping quality assessment dimensions. Although the adopted coding framework was refined iteratively and all included studies were revisited to refine the categorization, the reproducibility of the synthesis cannot be fully established. Future studies should consider independent coding by multiple reviewers or formal assessment of inter-rater agreement to strengthen the reproducibility of the findings.

The distinction between data quality and mapping quality also introduces ambiguity. For example, structurally valid FHIR outputs may still reflect incorrect mappings due to inappropriate transformations. This suggests that structural conformance is often interpreted as data quality, while dimensions such as coverage better capture mapping completeness.

Mapping approaches and quality assessment dimensions are highly dependent on the source data. Different formats and contexts (e.g., legacy HL7 data versus tabular data) introduce varying challenges and influence which quality dimensions are relevant.

Expert consultation provided preliminary feedback on the conceptual validity and practical relevance of the proposed framework but represents interpretive rather than empirical validation. The limited number of experts (*n* = 4) and contextual perspectives restrict generalizability, and further evaluation is needed. Given the small sample, the quantitative ratings were used only to describe individual expert perspectives and should not be interpreted as evidence of consensus among FHIR experts. The participating experts were primarily from European contexts, including the Nordic countries. Consequently, the consultation does not capture the full diversity of FHIR implementation practices internationally. In particular, implementation contexts such as the United States, where FHIR mapping is influenced by jurisdiction-specific implementation guides and regulatory requirements, were not represented. Furthermore, the purposive recruitment strategy may have introduced selection bias. The expert-informed requirements should therefore be regarded as preliminary and require further refinement and validation.

This study also has methodological limitations. The literature search was restricted to IEEE Xplore, ACM Digital Library, PubMed, and the JMIR journal family. Furthermore, only publications in the English language were considered and no systematic snowballing was conducted. Although this may have resulted in some relevant studies being missed, we believe that the included literature provided a representative overview of current FHIR mapping quality assessment practices. This interpretation is supported by the consistency between the literature findings, our own implementation experiences, and the preliminary results obtained through the FHIR expert consultation. The proposed framework remains conceptual and has not been empirically validated. Additionally, thematic analysis relies on subjective interpretation, no formal quality appraisal was performed, and distinctions between data and mapping quality were not always clear in the included studies.

## Conclusion

This review consolidates current preliminary evidence on tools, validation practices, and challenges related to assessing HL7 FHIR data mapping quality. Despite the growing adoption of FHIR, mapping quality assessment remains fragmented, largely manual, and insufficiently standardized. The findings support the need for a more formalized approach to pre-mapping assessment that can complement existing post-mapping conformance checks and improve transparency, consistency, and interoperability in FHIR mapping initiatives.
