# Supplementary material for: FHIR Data Mapping Quality Assessment: A Scoping Review and Expert-Informed Requirements for Future Pre-Mapping Frameworks
Source: Comput Struct Biotechnol J. 2026 Oct 22;35(2):0231. doi: 10.34133/csbj.0231 (PMC13687643; doi:10.34133/csbj.0231)
Supplement: Supplementary file 1 [file csbj.0231.f1.zip › renamed_b32a8.docx]

**Figure S1. Distribution of mapping quality dimensions across the included studies.** Identified mapping quality dimensions are shown for each of the 39 included studies based on the thematic classification presented in Table 4.
