# Supplementary material for: FHIR Data Mapping Quality Assessment: A Scoping Review and Expert-Informed Requirements for Future Pre-Mapping Frameworks
Source: Comput Struct Biotechnol J. 2026 Oct 22;35(2):0231. doi: 10.34133/csbj.0231 (PMC13687643; doi:10.34133/csbj.0231)
Supplement: Supplementary file 1 [file csbj.0231.f1.zip › Supplementary_material_2_revised_260916.docx]

**Table S1. Individual expert assessment of the proposed mapping quality dimensions**

**NR = Not relevant**

| Quality dimension | Expert | Relevance | Clarity | Completeness | Main qualitative feedback |
| --- | --- | --- | --- | --- | --- |
| Coverage | E1 | 5 | 4 | 4 | Expected coverage to include mapped elements represented through extensions; questioned how extensions are treated. |
|  | E2 | 4 | 3 | 5 | Good coverage may indicate appropriate resource selection; questioned whether coverage refers specifically to Core FHIR before extensions. |
|  | E3 | 5 | 5 | 5 | Not all source data necessarily need to be mapped; coverage should therefore not necessarily be maximized. |
|  | E4 | 3 | 4 | NR | Full coverage may not provide real-world value depending on the intended use case. |
| Completeness | E1 | 4 | 4 | 4 | No major concern reported. |
|  | E2 | 3 | 3 | 4 | Required fields may be populated with fixed values such as “Unknown,” complicating interpretation. |
|  | E3 | 5 | 5 | 3 | Suggested also considering terminology mappings, such as ConceptMaps for code systems/value sets. |
|  | E4 | 5 | 5 | NR | Questioned whether completeness is already addressed by existing validators. |
| Semantic correctness | E1 | 4 | 4 | 4 | No major concern reported. |
|  | E2 | 4 | 5 | 5 | Considered important; selected among the most critical dimensions. |
|  | E3 | 5 | 5 | 5 | Considered among the most critical dimensions. |
|  | E4 | 2 | 5 | NR | Questioned objective correctness because different FHIR representations may legitimately serve different use cases. |
| Terminology accuracy | E1 | 5 | 4 | 4 | Considered among the most critical dimensions. |
|  | E2 | 2 | 5 | 5 | Importance depends strongly on project-specific profiles; terminology mapping may be handled by a separate clinical team. |
|  | E3 | 5 | 5 | 4 | Suggested considering use of international standards instead of local codes where applicable. |
|  | E4 | 3 | 2 | NR | Definition was considered unclear and not fully aligned with the stated error example. |
| Structural conformance | E1 | 5 | 5 | 5 | Considered among the most critical dimensions. |
|  | E2 | 4 | 3 | 4 | Potential conceptual overlap with Coverage. |
|  | E3 | 5 | 4 | 5 | Considered among the most critical dimensions. |
|  | E4 | 4 | 3 | NR | Suggested that this is largely handled by existing FHIR validators and may not be mapping-specific. |
| Inter-rater reliability | E1 | 3 | 4 | 4 | — |
|  | E2 | 3 | 3 | 5 | Name considered confusing; disagreement may be expected because FHIR does not always have one correct representation. |
|  | E3 | 4 | 2 | 5 | Suggested renaming to **“Mapping concordance.”** |
|  | E4 | 4 | 4 | NR | Considered interesting but potentially overlapping with other dimensions. |
| Retrievability | E1 | 3 | 4 | 4 | — |
|  | E2 | 2 | 5 | 5 | Considered less problematic because custom search parameters can be defined. |
|  | E3 | 5 | 5 | 5 | Considered relevant without additional qualification. |
|  | E4 | NR | NR | NR | Relationship between retrievability and mapping quality was unclear to the expert. |

**Table S2. Main themes from expert feedback and implications for the proposed dimensions**

| Theme identified from expert feedback | Supporting expert feedback | Implication for interpretation/refinement |
| --- | --- | --- |
| Coverage is context-dependent | Full coverage may not always provide practical value; not all source data necessarily need to be mapped. | Coverage should be interpreted relative to the intended use rather than as a dimension that should always be maximized. |
| Extensions should not automatically indicate poor coverage | Experts questioned whether elements represented through extensions should count as covered and noted that extensions are expected within FHIR. | Clarify the distinction between overall FHIR coverage and reliance on Core FHIR versus extensions. |
| Semantic correctness may not have a single objective answer | Multiple FHIR representations may legitimately model the same information depending on the intended use. | Semantic correctness should account for intended use and implementation context rather than assume one universally correct mapping. |
| Terminology accuracy depends on implementation context | Experts emphasized project-specific profiles, international/local terminology standards, and jurisdiction-specific requirements. | Terminology assessment should consider profiles, IGs, terminology governance, and relevant national/international standards. |
| Structural conformance and completeness overlap with existing validation | Experts noted that existing FHIR validators can already detect many structural and completeness problems. | Position these dimensions as complementary to existing post-mapping validators rather than functions that a pre-mapping framework should replace. |
| Inter-rater reliability terminology may require refinement | “Inter-rater reliability” was considered confusing; “Mapping concordance” was proposed. | Clarify that the dimension concerns consistency/reproducibility between independent mapping decisions. |
| Retrievability has context-dependent relevance | One expert considered custom search parameters sufficient; another could not clearly connect retrievability to mapping quality, while another rated it highly relevant. | Retain the dimension but acknowledge that its relevance depends on downstream use and whether mapped data must support standardized retrieval. |
| FHIR lifecycle/maturity may influence mapping quality | Resource maturity and planned changes between FHIR releases were raised as additional considerations. | Include FHIR maturity/evolution as a consideration within FHIR-native assessment rather than necessarily creating a separate dimension. |
| Alignment with jurisdictional standards is important | National information models and terminologies may be more important than consistency with mappings developed elsewhere. | Explicitly incorporate implementation guides, local/national profiles, and governance context into FHIR-native assessment. |
| Source-data quality may affect mapping assessment | An expert suggested that the input dataset itself should also be assessed. | Maintain the conceptual distinction between source-data quality and mapping quality, while recognizing source quality as an important contextual factor. |

**Table S3. Individual expert assessment of the proposed pre-mapping framework requirements**

| Requirement presented to experts | Expert | Relevance | Feasibility | Main qualitative feedback |
| --- | --- | --- | --- | --- |
| Pre-mapping focus | E1 | 4 | 3 | — |
|  | E2 | 5 | 4 | Evaluation already occurs during design, sometimes unconsciously; systematic evaluation could support quality and explainability. |
|  | E3 | 2 | 3 | — |
|  | E4 | 5 | 3 | — |
| User-friendly | E1 | 3 | 2 | — |
|  | E2 | 3 | 4 | A user-friendly interface could support explanation to and involvement of clinical team members despite available FHIR/programming expertise. |
|  | E3 | 3 | 3 | — |
|  | E4 | 4 | 5 | — |
| Intuitive output | E1 | 4 | 3 | — |
|  | E2 | 4 | 5 | Suggested possible overlap with the User-friendly requirement. |
|  | E3 | 5 | 5 | — |
|  | E4 | 4 | 4 | — |
| FHIR-native alignment | E1 | 4 | 2 | — |
|  | E2 | 5 | 5 | — |
|  | E3 | 5 | 5 | Questioned the relevance of the 80/20 rule and FMM for profiling; suggested greater emphasis on profile quality. |
|  | E4 | 4 | 4 | Considered valuable as contextual information rather than a pure metric. |
| Comprehensive coverage of mapping quality dimensions | E1 | 4 | 2 | — |
|  | E2 | 4 | 5 | — |
|  | E3 | 4 | 4 | — |
|  | E4 | 3 | 3 | Noted that implementation may be feasible using LLM-based tools. |
| Flexible/adaptable across domains and datasets | E1 | 4 | 1 | — |
|  | E2 | 5 | 3 | — |
|  | E3 | 2 | 2 | — |
|  | E4 | 5 | 3 | — |
